# Evaluation of a Fully Automated Antinuclear Antibody Indirect Immunofluorescence Assay in Routine Use

**DOI:** 10.3389/fimmu.2020.607541

**Published:** 2020-12-04

**Authors:** Hyun-Woo Choi, Yong Jun Kwon, Ju-Heon Park, Seung-Yeob Lee, Sejong Chun, Eun Jeong Won, Jun Hyung Lee, Hyun-Jung Choi, Soo Hyun Kim, Myung-Geun Shin, Jong-Hee Shin, Seung-Jung Kee

**Affiliations:** ^1^ Department of Laboratory Medicine, Chonnam National University Bitgoeul Hospital, Gwangju, South Korea; ^2^ Department of Laboratory Medicine, Chonnam National University Hospital, Gwangju, South Korea; ^3^ Department of Laboratory Medicine, Chonnam National University Hwasun Hospital, Hwasun, South Korea; ^4^ Department of Laboratory Medicine, Chonnam National University Medical School, Gwangju, South Korea; ^5^ Department of Parasitology and Tropical Medicine, Chonnam National University Medical School, Gwangju, South Korea; ^6^ Department of Microbiology, Chonnam National University Medical School, Gwangju, South Korea

**Keywords:** antinuclear antibody, immunofluorescence assay, automation, pattern recognition, titer estimation

## Abstract

Indirect immunofluorescence assay (IFA) using HEp-2 cells as a substrate is the gold standard for detecting antinuclear antibodies (ANA) in patient serum. However, the ANA IFA has labor-intensive nature of the procedure and lacks adequate standardization. To overcome these drawbacks, the automation has been developed and implemented to the clinical laboratory. The purposes of this study were to evaluate the analytical performance of a fully automated Helios ANA IFA analyzer in a real-life laboratory setting, and to compare the time and the cost of ANA IFA testing before and after adopting the Helios system. A total of 3,276 consecutive serum samples were analyzed for ANA using the Helios system from May to August 2019. The positive/negative results, staining patterns, and endpoint titers were compared between Helios and visual readings. Furthermore, the turnaround time and the number of wells used were compared before and after the introduction of Helios system. Of the 3,276 samples tested, 748 were positive and 2,528 were negative based on visual readings. Using visual reading as the reference standard, the overall relative sensitivity, relative specificity, and concordance of Helios reading were 73.3, 99.4, and 93.4% (*κ* = 0.80), respectively. For pattern recognition, the overall agreement was 70.1% (298/425) for single patterns, and 72.4% (89/123) for mixed patterns. For titration, there was an agreement of 75.9% (211/278) between automated and classical endpoint titers by regarding within ± one titer difference as acceptable. Helios significantly shortened the median turnaround time from 100.6 to 55.7 h (*P* < 0.0001). Furthermore, routine use of the system reduced the average number of wells used per test from 4 to 1.5. Helios shows good agreement in distinguishing between positive and negative results. However, it still has limitations in positive/negative discrimination, pattern recognition, and endpoint titer prediction, requiring additional validation of results by human observers. Helios provides significant advantages in routine laboratory ANA IFA work in terms of labor, time, and cost savings. We hope that upgrading and developing softwares with more reliable capabilities will allow automated ANA IFA analyzers to be fully integrated into the routine operations of the clinical laboratory.

## Introduction

Antinuclear antibodies (ANA) are one of the most important serological markers used for the diagnosis of systemic autoimmune rheumatic diseases (SARD) such as systemic lupus erythematosus (SLE), systemic sclerosis (SSc), Sjögren’s syndrome (SjS), mixed connective tissue disease (MCTD), and idiopathic inflammatory myopathy (IIM). Steady increases in the prevalence of SARD have been reported in recent years, which has been attributed to a variety of causes, including exposure to environmental chemicals and toxins, an aging population and its associated chronic diseases, and use of particular drug regimens (1). With this increase in disease prevalence, the ANA test requests are increased by non-rheumatological clinicians to exclude SARD in patients due to the high negative predictive value of ANA measurement (2, 3).

Indirect immunofluorescence assay (IFA) using human epithelial cell tumor (HEp-2) cells is the most established method for ANA screening (4). The main benefits of the ANA IFA are the detection of wide-ranging autoantibodies, high sensitivity, and the possibility of concurrently determining staining patterns and titers (5). Nevertheless, the ANA IFA has several drawbacks, including the labor-intensive nature of the procedure and a lack of adequate standardization (5–7). Notably, pattern recognition, which depends on the individual abilities of investigators, can result in significant inter and intra-laboratory variabilities (8, 9). To overcome those challenges, several alternative techniques have been developed as potential replacements for IFA (i.e., single and multiplex immunometric assays, such as enzyme-linked immunosorbent assays, line immunoassays, and multiplex bead assays), promising improvements in standardization, throughput, and objectivity in results (10, 11). However, contrary to expectations, these alternative methods can vary significantly in sensitivity and diagnostic accuracy due to the difference in source, purity, concentration, binding capacity, and the limited number of antigens (10–13). Based on concerns regarding the newer assays and their associated limitations, the American College of Rheumatology (ACR) recommended IFA as the gold standard for ANA testing (14). In the context of standardization in ANA IFA testing and reporting, the International Consensus on ANA Patterns (ICAP) has been established, aiming to reach the consensus on nomenclature and definition of Hep-2 cell IFA patterns. The ICAP provides standardized categorization and nomenclature distinguishing different fluorescence patterns from AC (anti-cellular)-1 to AC-29, including AC-0 (negative), as well as interpretation guidelines of the 29 distinct patterns (15–18). In addition to such increased demand for ANA testing and standardization efforts, the automation of slide preparation, image acquisition, titration, and interpretation were developed and evaluated for implementation to the clinical laboratory (8, 19–29).

Among the commercial automated systems, Helios (Aesku Diagnostics, Wendelsheim, Germany) is the only fully automated IFA processor in which the automated digital image acquisition and ANA reading systems are integrated with slide processing in one instrument (2, 8). During the full process, no intervention is needed, offering users a true hands-off time. The system employs barcode readers for complete traceability, a unique three needle system for fast pipetting operations enabling non-stop performance, a motorized and autofocus fluorescence microscope, and specially designed software using mathematical algorithms for discrimination of positive and negative results, identification of ANA patterns and titers.

By integrating the fully automatic ANA IFA analyzer in our laboratory, we aimed to establish a fast and efficient workflow for ANA testing. Here, we evaluated the performance of the Helios system in our real-life laboratory setting, where patient groups are less clearly defined, and test orders are not based on pre-defined criteria. Additionally, we compared the time and the cost of ANA IFA testing before and after adopting the Helios system.

## Materials and Methods

### Sample Collection

**Figure 1:**
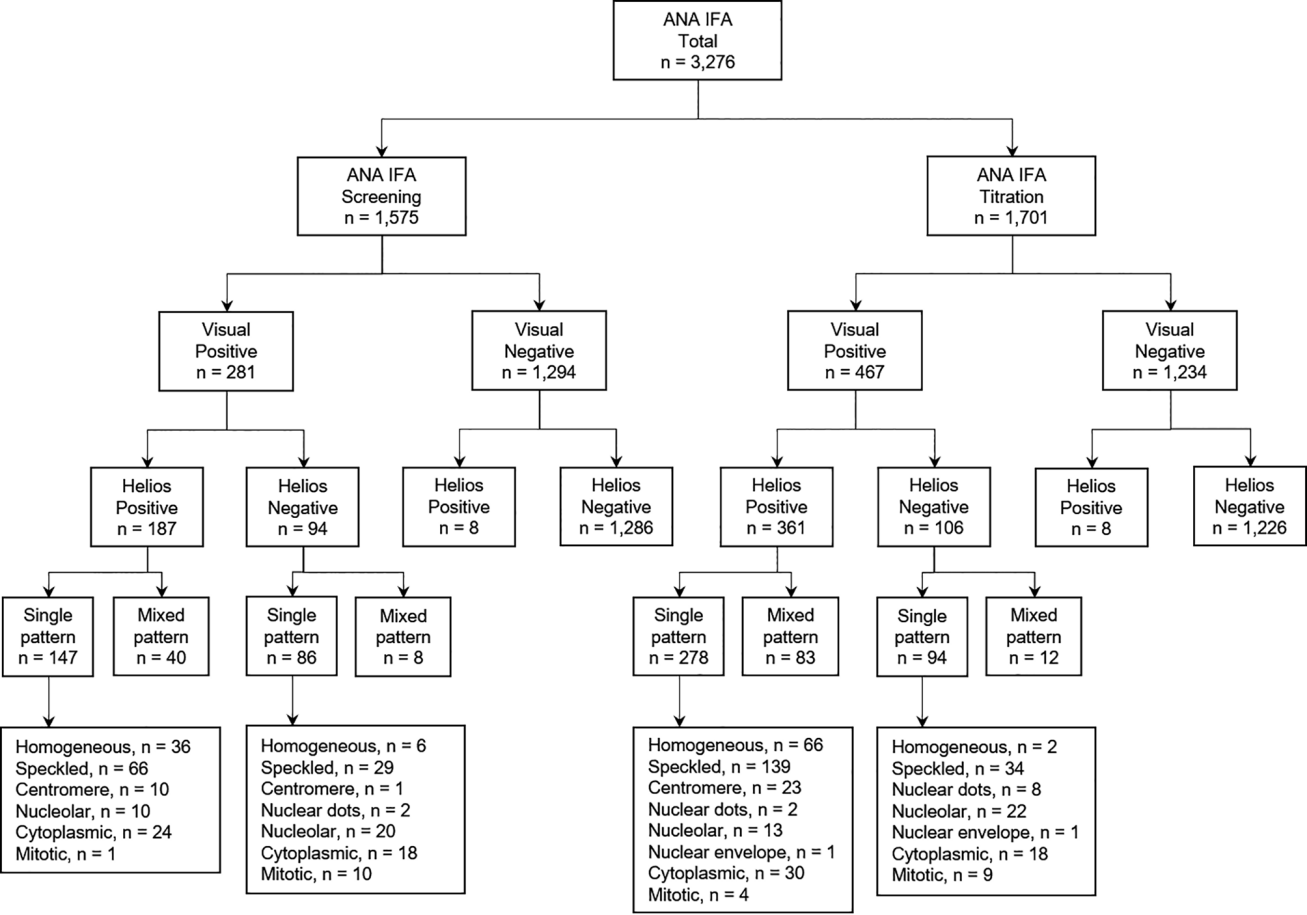
Study design and sample flowchart for evaluation of Helios automated ANA IFA analyzer. ANA, antinuclear antibody; IFA, indirect immunofluorescence assay; n, number.

Between May and August 2019, a total of 3,276 consecutive serum samples obtained from 3,164 patients were referred for routine ANA testing to the Diagnostic Immunology Laboratory at Chonnam National University Hospital, Gwangju, South Korea. The study design and sample flowchart are described in **Figure 1**. This study was approved by the Institutional Review Board of Chonnam National University Hospital (IRB CNUH-2019-304). Due to the nature of this study, the Institutional Review Board of Chonnam National University Hospital waived the requirement for informed consent.

### Automated ANA IFA

ANA tests were performed on a Helios automated analyzer using the ANA HEp-2 standard kit and Helios software version 3.1 (Aesku Diagnostics) according to the manufacturer’s instructions. Briefly, serum samples were loaded in the Helios system, and the tests were automatically conducted at 1:80 dilution. Digital images are taken by a camera and stored on the computer system. The positive/negative classification module leverages the image features such as the structure of the objects, the fluorescence signal intensity (FI), and the background/cell ratio (8). The cut-off value of FI was 70. Three images were taken for each sample, and samples with two or more images classified as positive were defined as ‘positive’. For positive pre-classified samples, the software tool of the Helios system recognizes the pattern of the captured image by using SVM (Support Vector Machine) algorithm. The system also provides automatically predicted endpoint titers based on the measured FI. Since the Helios software has not accommodated the ICAP classification yet, it reports staining patterns as following: homogeneous, speckled, centromere, nuclear dots, nucleolar, nuclear envelope, and cytoplasmic (22, 23).

After all automated procedures, two experienced observers initially interpreted the stored digital images independently without knowledge of the suggested interpretation of Helios, and if the two experts disagreed, a consensus was reached by discussion. As recommended by ICAP, we endeavor to report all 29 HEp-2 cell IFA patterns in standardized nomenclature. To compare the patterns by visual reading with Helios reading, we assigned AC-1 as homogeneous; AC-2, AC-4, AC-5, AC-29 as speckled; AC-3 as centromere; AC-6, AC-7 as nuclear dots; AC-8, AC-9, AC-10 as nucleolar; AC-11, AC-12 as nuclear envelope; AC-15 to AC-23 as cytoplasmic; and AC-13, AC-14, AC-24 to AC-28 as others. In case of the samples referred for screening tests only, positive samples were not proceeded with any further dilution and reported as positive with patterns. In case of the samples referred for titration tests, positive samples identified using the standard 1:80 dilution in the screening mode were further diluted. The classical endpoint titers based on the visual reading of the images from serial dilution were reported with patterns. For quality control, two standards (one positive and one negative) provided in the test kit, and two patient serum samples [one positive having a homogeneous (AC-1) pattern with a titer of 1:320 and one negative (AC-0)] were tested in parallel.

### Statistical Analysis

By using visual expert reading as a standard, true positive (TP) was defined as visual-positive and Helios-positive; false positive (FP) as visual-negative and Helios-positive; false negative (FN) as visual-positive and Helios-negative; and true negative (TN) as visual-negative and Helios-negative. Relative sensitivity was calculated as TP/(TP + FN) × 100, and relative specificity was calculated as TN/(TN + FP) × 100. The degree of concordance between Helios and visual readings was assessed by overall agreement (*oa*) percentage or by Cohen’s *κ*. The *oa* percentage was calculated as (TP + TN)/(TP + FP + FN + TN) × 100. Cohen’s *κ* values is defined as (*oa* - *ca*)/(1 - *ca*), where *ca* is hypothetical probability of chance agreement calculated as [(TP + FP)(TP + FN) + (FN + TN)(FP + TN)]/(TP + FP + FN + TN)^2^. Cohen’s *κ* values were interpreted as follows: ≤ 0.20 as poor, 0.21–0.40 as fair, 0.41–0.60 as moderate, 0.61–0.80 as good, and 0.81–1.00 as very good agreement (30). Fisher’s exact test was used for comparison of proportions. The turnaround times (TATs) were defined as follows: TAT_[1]_, the time from blood sampling to sample receipt; TAT_[2]_, the time from sample receipt to results reporting; and TAT_[Total]_, the time from blood sampling to results reporting. Normality test for distribution of age and TATs was performed by D’Agostino-Pearson test. Mann-Whitney *U* test was used to compare TATs before and after the use of the Helios system. All statistical analyses were performed using R software version 3.6.1, and graphics were prepared using GraphPad Prism software version 6.0. P values < 0.05 were considered statistically significant.

## Results

### Study Population

The clinical and demographic characteristics of patients subjected to ANA testing are summarized in **Table 1**. A total of 3,276 consecutive serum samples were obtained from 3,164 patients [60.4% female; median age (interquartile range), 53.7 (37.9–64.1) years]. Single, two, and three samples per patient were collected from 3,057 (96.6%), 102 (3.2%), and 5 (0.2%) patients, respectively. The ANA tests were requested by the Departments of Rheumatology (44.9%), Internal Medicine other than Rheumatology (25.5%), Dermatology (11.6%), Neurology (6.6%), Pediatrics (5.7%), and others (5.7%).

**Table 1:** Clinical and demographic characteristics of consecutive patients referred for ANA IFA tests.

Characteristics	Patient		Sample					
Total number	3,164		3,276					
Sex, *n* (%)								
Female	1,910 (60.4)							
Male	1,254 (39.6)							
Age, year								
Median (IQR)^a^	53.7 (37.9–64.1)							
Range	0.4-97.0							
Patients with multiple given samples, *n* (%)								
One sample	3,057	(96.6)	3,057			(93.3)		
Two samples	102	(3.2)	204			(6.2)		
Three samples	5	(0.2)	15			(0.5)		
Purpose of request according to department, *n* (%)^b^			Screening		Titration		Total	
Rheumatology			370	(23.5)^c^	1,101	(64.7)^c^	1,471	(44.9)
Internal medicine			348	(22.1)^d^	488	(28.7)^d^	836	(25.5)
Dermatology			363	(23.0)^e^	17	(1.0)^e^	380	(11.6)
Neurology			191	(12.1)^f^	24	(1.4)^f^	215	(6.6)
Pediatrics			175	(11.1)^g^	12	(0.7)^g^	187	(5.7)
Others			128	(8.1)^h^	59	(3.5)^h^	187	(5.7)
Total			1,575	(48.1)^i^	1,701	(51.9)^i^	3,276	(100)

a Normality test for distribution of age was performed by D’Agostino-Pearson test, showing non-Gaussian distributions of age (P < 0.0001).

^b^P values for comparison of proportions of request departments between screening and titration were calculated using Fisher’s exact test. Values with the same superscript lowercase letters were compared with each other: ^c^P < 0.0001; ^d^P < 0.0001; ^e^P < 0.0001; ^f^P < 0.0001; ^g^P < 0.0001; ^h^P < 0.0001; and ^i^P = 0.0021.

ANA, antinuclear antibody; IFA, indirect immunofluorescence assay; n, number; and IQR, interquartile range.

### Positive/Negative Discrimination

The analytical performance of Helios automated reading for discriminating between positive and negative ANA results is summarized in **Table 2**. Among a total of 3,276 samples, visual reading yielded 748 (22.8%) positive and 2,528 (77.2%) negative results. Of the 748 positive samples by visual reading, 548 (73.3%) were positive and 200 (26.7%) negative by Helios reading. Of the 2,528 negative samples by visual reading, 16 (0.6%) were positive and 2,512 (99.4%) negative by Helios reading. Using visual reading as the reference standard, the overall relative sensitivity, relative specificity, and concordance of Helios reading were 73.3, 99.4, and 93.4% (*κ* = 0.80), respectively.

**Table 2:** Analytical performance of Helios automated ANA IFA analyzer for positive/negative discrimination.

Heterogeneity factor	Total	TP^a^	FP^a^	FN^a^	TN^a^	Analytical performance of Helios			
						Relative sensitivity% (95% CI)	Relative specificity% (95% CI)	Concordance	
								Agreement (%)	Cohen’s *κ* (95% CI)
Overall	3,276	548	16	200	2,512	73.3 (69.9–76.4)	99.4 (99.0–99.6)	93.4	0.80 (0.77–0.82)
Screening^b^	1,575	187	8	94	1,286	66.6 (60.7–72.0)	99.4 (98.8–99.7)	93.5	0.75 (0.70–0.80)
Titration^b^	1,701	361	8	106	1,226	77.3 (73.2–81.0)	99.4 (98.7–99.7)	93.3	0.82 (0.79–0.85)
						*P* = 0.0016^c^			
≥ 1:80^d^	1,701	361	8	106	1,226	77.3 (73.2–81.0)	99.4 (98.7–99.7)	93.3	0.82 (0.79–0.85)
≥ 1:160^d^	1,701	294	1	13	1,393	95.8 (92.9–97.7)	99.9 (99.6–100)	99.2	0.97 (0.96–0.99)
						*P* < 0.0001	*P* = 0.0156		*P* < 0.0001

a TP, FP, FN, TN are defined by the visual reading used as a standard.

b Prevalence of ANA-positive results [(TP + FN)/Total] was significantly higher in titration samples compared with screening samples (27.5% vs. 17.8%, P < 0.0001).

c All P values for comparison of proportions were calculated using Fisher’s exact test. P < 0.05 was considered significant.

d According to inclusion or exclusion of weak positive, the ANA-positive sample was defined as one with a titer of ≥ 1:80 or one with a titer of ≥ 1:160.

ANA, antinuclear antibody; CI, confidence interval; FN, false negative; FP, false positive; IFA, indirect immunofluorescence assay; TN, true negative: and TP, true positive.

Of the total samples requested for ANA testing, 1,575 were assigned for screening and 1,701 for titration (**Figure 1**). The relative sensitivity of Helios reading was found to be significantly higher in samples requested for titration compared with screening (77.3 vs. 66.6%, *P* < 0.005; **Table 2**). To investigate the impact of the inclusion of weakly positive samples on analytical performance of Helios reading, we compared the analytical performance between inclusion and exclusion of weakly positive samples in samples requested for titration. The relative sensitivity, relative specificity, and concordance of Helios reading were found to be significantly higher in samples with titers ≥ 1:160 compared with titers ≥ 1:80 (95.8 vs. 77.3%, *P* < 0.0001; 99.9 vs. 99.4%, *P* < 0.05; 99.2% (*κ* = 0.97) vs. 93.3% (*κ* = 0.82), *P* < 0.0001, respectively; **Table 2**).

### Discrepancy Analysis

Discrepancies between Helios and visual readings for positive/negative discrimination are summarized in **Table 3**. Among a total of 200 false negative samples, only 106 were referred for titration tests. The titration data revealed that 105 (99.1%) had a titer of ≤ 1:160, and the remaining one (0.9%) had a titer of 1:320 with an intercellular bridge pattern (AC-27). These samples were derived from patients with SLE (*n* = 8), SjS (*n* = 4), rheumatoid arthritis (RA; *n* = 13), autoimmune hepatitis (*n* = 1), infection (*n* = 12), and other conditions (*n* = 68). Of the 16 false positive samples, all samples had a automated endpoint titer of ≤ 1:160, and were derived from patients with SjS (*n* = 1), infection (*n* = 3), malignancy (*n* = 2), RA (*n* = 2), neuropathy (*n* = 1), skin disease (*n* = 5), proteinuria (*n* = 1), and cerebral infarction (*n* = 1).

**Table 3:** Discrepancy analysis of positive/negative discrimination between Helios and visual readings according to ANA pattern and titer.

Helios		Visual		*n* (%)	Endpoint titer, *n* (%)		
Positive/Negative	Pattern	Positive/Negative	Pattern^a^		1:80	1:160	1:320
False negative^b^				106 (100)^b^	93 (87.7)	12 (11.3)	1 (0.9)
Negative		Positive	Homogeneous	2 (1.9)	2 (100)	–	–
Negative		Positive	Speckled	34 (32.1)	31 (91.2)	3 (8.8)	–
Negative		Positive	Nuclear dots	8 (7.5)	8 (100)	–	–
Negative		Positive	Nucleolar	22 (20.8)	20 (90.9)	2 (9.1)	–
Negative		Positive	Nuclear envelope	1 (0.9)	–	1 (100)	–
Negative		Positive	Cytoplasmic	18 (17.0)	18 (100)	–	–
Negative		Positive	Others	9 (8.5)	7 (77.8)	1 (11.1)	1 (11.1)
Negative		Positive	Mixed	12 (11.3)	7 (58.3)	5 (41.7)	–
False positive^c^				16 (100)^c^	15 (93.8)	1 (6.3)	–
Positive	Speckled	Negative		11 (68.8)	10 (90.9)	1 (9.1)	–
Positive	Cytoplasmic	Negative		1 (6.3)	1 (100)	–	–
Positive	Unknown	Negative		4 (25.0)	4 (100)	–	–

a The patterns interpreted by visual reading were classified in ICAP nomenclature. In this study, to compare the patterns between Helios and visual readings, we assigned AC-1 as homogeneous; AC-2, AC-4, AC-5, AC-29 as speckled; AC-3 as centromere; AC-6, AC-7 as nuclear dots; AC-8, AC-9, AC-10 as nucleolar; AC-11, AC-12 as nuclear envelope; AC-15 to AC-23 as cytoplasmic; and AC-13, AC-14, AC-24 to AC-28 as others.

b Among a total of 200 false negative samples, only 106 samples were referred for titration. Here the classical endpoint titers obtained by serial dilution are stated.

c For false positive samples, the stated endpoint titers are automated endpoint titers obtained by Helios software.

ANA, antinuclear antibody; n, number.

### Pattern Recognition

For samples showing single patterns by both Helios and visual readings, the overall agreement between Helios and visual readings was 70.1% (298/425)(**Table 4**). The agreement rates for individual patterns were as follows: 77.5% (79/102) for homogeneous, 73.7% (151/205) for speckled, 69.7% (23/33) for centromere, 100% (2/2) for nuclear dots, 91.3% (21/23) for nucleolar, 0% (0/1) for nuclear envelope, and 40.7% (22/54) for cytoplasmic. Helios system incorrectly identified 18.6% (19/102) of homogeneous as speckled; 22.9% (47/205) of speckled as homogeneous; and 30.3% (10/33) of centromere as speckled. Cytoplasmic patterns were incorrectly identified as various patterns.

**Table 4:** Agreement between Helios and visual readings for pattern recognition in samples with single pattern.

Visual^a^	Helios								Agreement (%)
	Homogeneous	Speckled	Centromere	Nucleardots	Nucleolar	Nuclearenvelope	Cytoplasmic	Unknown	
Homogeneous (*n* = 102)	**79** ^b^	19	–	–	–	–	1	3	77.5
Speckled (*n* = 205)	47^c^	**151**	–	–	2	1	3	1	73.7
Centromere (*n* = 33)	–	10	**23**	–	–	–	–	–	69.7
Nuclear dots (*n* = 2)	–	–	–	**2**	–	–	–	–	100
Nucleolar (*n* = 23)	–	1	–	–	**21**	–	–	1	91.3
Nuclear envelope (*n* = 1)	–	–	–	–	–	–	1	–	0
Cytoplasmic (*n* = 54)	–	6	1	2	11	–	**22**	12	40.7
Others (*n* = 5)	2	2	–	–	–	–	–	1	NA
Total (*n* = 425)	128	189	24	4	34	1	27	18	**70.1** ^d^

a To compare the patterns between Helios and visual readings, we assigned AC-1 as homogeneous; AC-2, AC-4, AC-5, AC-29 as speckled; AC-3 as centromere; AC-6, AC-7 as nuclear dots; AC-8, AC-9, AC-10 as nucleolar; AC-11, AC-12 as nuclear envelope; AC-15 to AC-23 as cytoplasmic; and AC-13, AC-14, AC-24 to AC-28 as others.

b Number of the concordant results are emphasized in bold.

c Included AC-2 (n = 35), AC-4 (n = 2), and AC-5 (n = 10).

d κ = 0.61 calculated by using data from only 7 patterns, including homogeneous, speckled, centromere, nuclear dots, nucleolar, nuclear envelope, and cytoplasmic patterns, as unknown patterns by Helios and other patterns by visual are not identical.

AC, anticellular; n, number; NA, not available.

For samples showing mixed patterns, the overall agreement between Helios and visual readings was 72.4% (89/123)(**Table 5**). As the Helios software can suggest only one pattern, if the suggested pattern was one of the mixed patterns by visual reading, it was considered concordant.

**Table 5:** Agreement between Helios and visual readings for pattern recognition in samples with mixed pattern.

Visual^a^	Helios								Agreement(%)
	Homogeneous	Speckled	Centromere	Nucleardots	Nucleolar	Nuclearenvelope	Cytoplasmic	Unknown	
Speckled/Cytoplasmic (*n* = 22)		**13** ^b^					**6**	3	86.4
Speckled/Nuclear dots (*n* = 21)	4	**9**		**6**				2	71.4
Nucleolar/Cytoplasmic (*n* = 14)		1			**2**		**9**	2	78.6
Homogeneous/Speckled (*n* = 10)	**2**	**8**							100
Homogeneous/Cytoplasmic (*n* = 10)	**1**	5					**4**		50.0
Homogeneous/Nucleolar (*n* = 9)	**1**	6			**2**				33.3
Speckled/Nucleolar (*n* = 6)		**3**			**3**				100
Nuclear dots/Cytoplasmic (*n* = 6)				**1**		1	**4**		83.3
Homogeneous/Nuclear dots (*n* = 4)		1		**3**					75.0
Centromere/Cytoplasmic (*n* = 3)		1	**1**				**1**		66.7
Cytoplasmic/Others (*n* = 2)							**2**		100
Centromere/Nuclear envelope (*n* = 2)		2							0
Nuclear envelope/Cytoplasmic (*n* = 2)							**2**		100
Homogeneous/Centromere (*n* = 1)		1							0
Speckled/Centromere (*n* = 1)		**1**							100
Centromere/Nucleolar (*n* = 1)					**1**				100
Nuclear dots/Nuclear envelope (*n* = 1)							1		0
Nuclear envelope/Others (*n* = 1)						**1**			100
Homogeneous/Speckled/Nucleolar (*n* = 1)		**1**							100
Homogeneous/Centromere/Cytoplasmic (*n* = 1)		1							0
Homogeneous/Nuclear dots/Cytoplasmic (*n* = 1)							**1**		100
Homogeneous/Nucleolar/Others (*n* = 1)		1							0
Speckled/Nuclear dots/Cytoplasmic (*n* = 1)		**1**							100
Nuclear dots/Cytoplasmic/Others (*n* = 1)					1				0
Cytoplasmic/Others/Others (*n* = 1)								1	NA
Total (*n* = 123)	8	55	1	10	9	2	30	8	**72.4**

a To compare the patterns between Helios and visual readings, we assigned AC-1 as homogeneous; AC-2, AC-4, AC-5, AC-29 as speckled; AC-3 as centromere; AC-6, AC-7 as nuclear dots; AC-8, AC-9, AC-10 as nucleolar; AC-11, AC-12 as nuclear envelope; AC-15 to AC-23 as cytoplasmic; and AC-13, AC-14, AC-24 to AC-28 as others.

b Number of the concordant results are emphasized in bold.

n, number; NA, not available.

### Endpoint Titer Estimation

**Figure 2:**
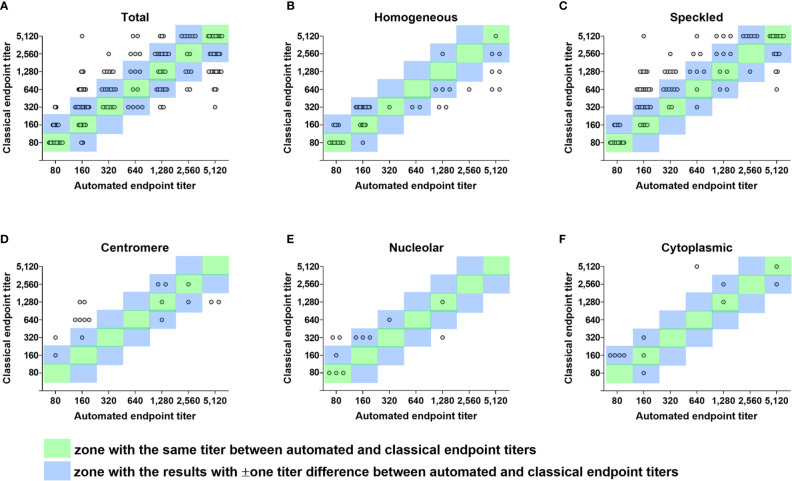
Error grid analysis of concordance between automated and classical endpoint titers. Differences between automated endpoint titer by Helios reading and classical endpoint titer by serial dilution among true positive samples with single pattern referred for ANA titration test only are shown in error grid and scatter plots. **(A)** A total of 278 true positive samples with single pattern. **(B)** 51 samples with the same homogeneous pattern by Helios and visual reading. **(C)** 106 samples with the same speckled pattern by Helios and visual reading. **(D)** 17 samples with the same centromere pattern by Helios and visual reading. **(E)** 12 samples with the same nucleolar pattern by Helios and visual reading. **(F)** 12 samples with the same cytoplasmic pattern by Helios and visual reading. Each circle represents an individual sample. Circles in the green zone indicate samples with the same titer between automated and classical endpoint titers. Circles in the blue zone indicate samples with ± one titer difference between automated and classical endpoint titers. Circles outside the two zones indicate error results with > ± one titer difference. ANA, antinuclear antibody.

For samples showing single patterns by both Helios and visual readings, by regarding within ± one titer difference as acceptable, the overall agreement between automated and classical endpoint titers was 75.9% (211/278) (**Table 6** and **Figure 2A**). The agreement rates between automated and classical endpoint titers, categorized as 1:80, 1:160, 1:320, 1:640, 1:1,280, 1:2,560, and 1:5,120 were as follows: 100% (57/57), 100% (44/44), 81.8% (45/55), 40.0% (16/40), 32.4% (11/34), 83.3% (20/24), and 75.0% (18/24), respectively.

**Table 6:** Agreement between automated and classical endpoint titers in 278 samples showing single pattern by both Helios and visual readings.

Classical endpoint titer	*n*	Automated endpoint titer, *n*							Agreement^a^(%)
		1:80	1:160	1:320	1:640	1:1,280	1:2,560	1:5,120	
1:80	57	**52** ^b^	**5**	–	–	–	–	–	100
1:160	44	**25**	**19**	–	–	–	–	–	100
1:320	55	6	**35**	**6**	**4**	3	–	1	81.8
1:640	40	–	19	**7**	**2**	**7**	2	3	40.0
1:1,280	34	–	7	5	**3**	**5**	**3**	11	32.4
1:2,560	24	–	–	1	3	**9**	**2**	**9**	83.3
1:5,120	24	–	1	–	2	3	**6**	**12**	75.0
Total	278	83	86	19	14	27	13	36	**75.9** ^c^

a Included the results within ± one titer difference which were deemed concordant.

b Number of the concordant results are emphasized in bold.

c κ = 0.23, reflecting only samples with the same titer between automated and classical endpoint titers.

n, number.

Concordance and error rates of automated endpoint titer were analyzed according to ANA pattern and the degree of titer difference in a total of 200 samples showing the same pattern by both Helios and visual readings (**Table 7** and **Figures 2B–F**). Of these samples, 60 (30.0%) had the same titer, 148 (74.0%) were within ± one titer difference, and 52 (26.0%) had more than ± one titer difference. In error results with more than ± one titer difference, automated endpoint titers of homogeneous patterns were significantly higher than classical endpoint titers (*P* < 0.0001), whereas those of speckled patterns were significantly lower (*P* < 0.01). The titer agreement for individual patterns, presented in descending order, were as follows: cytoplasmic (91.7%) > homogeneous (86.3%) > nucleolar (75.0%) > speckled (71.7%) > centromere (47.1%). Cross-tabulated data about automated and classical endpoint titers for individual patterns are presented in **Supplementary Tables 1–5**.

**Table 7:** Concordance and error rates of automated endpoint titer according to ANA pattern and the degree of titer difference.

Pattern^a^	*n*	Difference of automated endpoint titer from classical endpoint titer, *n* (%)											
		Concordant (≤ ±one titer)						Error (> ±one titer)					
		Same titer		±one titer		Total		Higher		Lower		Total	
Total	200	60	(30.0)	88	(44.0)	148	(74.0)	13	(6.5)	39	(19.5)	52	(26.0)
Homogeneous	51	17	(33.3)	27	(52.9)	44	(86.3)	7	(13.7)^b^	0	(0.0)^b^	7	(13.7)
Speckled	106	34	(32.1)	42	(39.6)	76	(71.7)	3	(2.8)^c^	27	(25.5)^c^	30	(28.3)
Centromere	17	2	(11.8)	6	(35.3)	8	(47.1)	2	(11.8)^d^	7	(41.2)^d^	9	(52.9)
Nuclear dots	2	0	(0.0)	0	(0.0)	0	(0.0)	0	(0.0)^e^	2	(100)^e^	2	(100)
Nucleolar	12	4	(33.3)	5	(41.7)	9	(75.0)	1	(8.3)^f^	2	(16.7)^f^	3	(25.0)
Cytoplasmic	12	3	(25.0)	8	(66.7)	11	(91.7)	0	(0.0)^g^	1	(8.3)^g^	1	(8.3)

^a^Only samples with the same single pattern by both Helios and visual readings were included. To compare the patterns between Helios and visual readings, we assigned AC-1 as homogeneous; AC-2, AC-4, AC-5, AC-29 as speckled; AC-3 as centromere; AC-6, AC-7 as nuclear dots; AC-8, AC-9, AC-10 as nucleolar; AC-11, AC-12 as nuclear envelope; AC-15 to AC-23 as cytoplasmic; and AC-13, AC-14, AC-24 to AC-28 as others.

^b^P values for comparison of proportions between higher and lower predicted automated endpoint titers were calculated using Fisher’s exact test. Values with the same superscript lowercase letters were compared with each other: ^b^P < 0.0001; ^c^P < 0.01; ^d^P > 0.05; ^e^P > 0.05; ^f^P > 0.05; and ^g^P > 0.05.

ANA, antinuclear antibody; n, number.

### Time and Cost Analysis

TAT and reagent consumption before and after the adoption of the Helios system in routine clinical practice were compared (**Table 8**). Our data showed that the median total TAT was significantly shortened from 100.6 h to 55.7 h after the introduction of Helios (*P* < 0.0001). Moreover, routine use of the Helios system also reduced the consumption of slide wells per test from 4 to 1.5.

**Table 8:** TAT and reagent consumption for ANA tests before and after the routine use of Helios system.

Parameter	Routine use of Helios system		*P* ^a^
	Before	After	
Total number	3,054	3,276	
Study period	May to August 2018	May to August 2019	
TAT_[1]_ ^b^, h			
Median (IQR)	0.6 (0.2–1.0)	0.6 (0.2–0.9)	<0.0001
TAT_[2]_ ^c^, h			
Median (IQR)	99.1 (61.8–123.8)	53.7 (30.7–99.0)	<0.0001
TAT_[*Total*]_ ^d^, h			
Median (IQR)	100.6 (64.7–124.5)	55.7 (31.7–99.4)	<0.0001
Screening, *n*	1,547	1,575	
HEp-2 slide wells	1,547	1,575	
Wells/test	1	1	
Titration, *n*	1,507	1,701	
HEp-2 slide wells	6,028	2,620	
Slide wells/test	4	1.5	

a P values for comparison of medians of two TATs were calculated using Mann-Whitney U test. Normality test for distribution of TATs was performed by D’Agostino-Pearson test, showing non-Gaussian distributions of TATs (P < 0.0001).

b The TAT_[1]_ was defined as the time from blood sampling to sample receipt.

c The TAT_[2]_ was defined as the time from sample receipt to results reporting.

d The TAT_[Total]_ was defined as the time from blood sampling to results reporting.

ANA, antinuclear antibody; h, hour; IQR, interquartile range; n, number; and TAT, turnaround time.

## Discussion

To the best of our knowledge, this is the most extensive single-center investigation assessing the performance, titration capability, TAT, and cost-effectiveness of Helios, a fully automated analyzer used for daily ANA IFA testing in a large set of consecutive patients with suspected SARD in a real-life setting. In this study, the overall relative sensitivity, relative specificity, and concordance of Helios reading was 73.3, 99.4, and 93.4% (*κ* = 0.80), respectively, which varied considerably from values obtained in several previous studies using various automated analyzers (23–26). The analytical performance of automated systems is significantly affected by factors such as sample selection bias, prevalence, inclusion rate of weakly positive samples, and the individual device being tested (8, 22, 31). Our subgroup analysis showed that the relative sensitivity and concordance with visual assessments were superior in titration samples compared with screening samples (**Table 2**). This observation is consistent with a previous study comparing samples processed at university and private laboratories (27). In screening samples, a low prevalence of SARD is usually expected (32). Our observation supported this expectation that the proportion of samples requested by the department of rheumatology showed a significantly higher percentage of titration samples compared with screening samples (64.7 versus 23.5%, *P* < 0.0001; **Table 1**). Additional analysis regarding weakly positive samples demonstrated that the analytical performance was better in cohorts with a low proportion of weakly positive samples than with a high proportion of weakly positive samples, consistent with previous results (28). This is supported by our observation that excluding weakly positive samples improves the concordance (Cohen’s *κ*) from 0.82 to 0.97.

In the present study, among a total of 3,276 samples, Helios mistakenly identified 200 (6.1%) as false negatives and 16 (0.5%) as false positives, suggesting that Helios missed a considerable number of visually positive cases. The main reason for this higher proportion of false negatives may be due to the inclusion of more samples with borderline FI from consecutive patients with suspected SARD than from well-defined patient groups. Previous studies also reported that automated ANA IFA systems have difficulties in differentiating negative and weakly positive samples (8, 20, 22, 33). This notion is consistent with our data showing that almost all false negatives had low titers (1:80 or 1:160). Recently, the ACR and the European League Against Rheumatism (EULAR) released new SLE criteria based on a scoring system including a positive ANA at a titer ≥ 1:80 by IFA occurring at least once as an entry criterion to ensure high sensitivity (34, 35). Our data showed that 8 of 106 false negative samples referred for titration missed by Helios were derived from SLE patients (**Supplementary Table 6**). This implies that the performances observed during routine laboratory use of the automated systems are not yet satisfactory. We further investigated the system’s positive/negative discrimination parameters regarding such false negative samples. Interestingly, 34% (68/200) had at least one image over the FI cut-off of 70, including 5 of 8 SLE samples. To avoid missing such cases, it would be helpful to check each image’s FI on the user interpretation module. Besides, adjusting the cut-off values could further increase the sensitivity of automated systems (26).

Our study showed that the overall concordance rates of pattern recognition between Helios and visual readings were 70.1% for single patterns and 72.4% for mixed patterns, which were similar to Daves et al.’s data (23). However, these values were lower than those in other previous studies (83.7–92.3%) (24, 26, 29). Such variation among studies may be due to the difference in the automated systems and reagents being used. Furthermore, our data showed that concordance rates varied from 0 to 100% according to individual patterns. The Helios system correctly recognized over 70% of homogeneous, speckled, centromere, nucleolar, and nuclear dots patterns, but less than 50% of nuclear envelope and cytoplasmic patterns, which, except for cytoplasmic patterns, is in line with previous studies (23–25, 29). Our data revealed that Helios incorrectly identified 22.9% (47/205) of speckled pattern as homogeneous. By further investigation, of these 47 cases, 35 (74.5%) were nuclear dense fine speckled (AC-2) pattern. Data from an international internet-based survey reported that AC-2 pattern was recognized with significantly lower accuracy and most often confused with homogeneous or other speckled patterns (36). Therefore, it is necessary to make a careful review of homogeneous patterns suggested by the Helios system. Collectively, these findings indicate that the analytical performance of Helios for pattern recognition is not fully satisfactory from a perspective of routine laboratory practice, still requiring expert intervention for a considerable number of assigned patterns.

Considering results within ± one titer difference as acceptable, the overall agreement between automated and classical endpoint titer was 75.9% (**Table 6**), consistent with previous studies (23, 24, 29). For samples with the same single patterns by both Helios and visual readings (**Table 7**), the overall error rate was 26.0%, similar to previous reports (25, 29, 37, 38). The high error rates may be due to the single-well titration method that most automated systems currently use. Won suggested the multi-well (2 or 3 wells) based line slope titration method to improve the accuracy (37). Moreover, our analysis on pattern dependency of automated endpoint titer prediction demonstrated that Helios predicted higher titers for homogeneous patterns but lower titers for speckled patterns, consistent with a previous study (37). The centromere pattern (AC-3), which possesses a lower overall fluorescence than the other common patterns, had a lower concordance rate of 47.1%, and most of the error results had lower titers (41.2%), in line with Zeng et al (25). We speculate that these phenomena are due to the different total amounts of fluorescent signals measured according to patterns. Taken together, these findings suggested the use of pattern-specific cut-off values or multi-well titration method to increase the accuracy of automated endpoint titer results.

In addition to the analytical performance of the automated system, hands-on time and material cost are essential concerns in a routine clinical laboratory. Helios shortened the TAT to nearly half of that seen using manual methods and decreased the number of slide wells used by two-thirds by adopting automated endpoint titer predictions as a guide before performing titer evaluation. Before implementing the Helios system in our laboratory, the workflow from sample preparation to results required approximately two working days, limiting our ability to perform ANA testing to two or three times per week. After the introduction of the Helios system, the ANA IFA test could be performed every working day. For well count saving, before the introduction of the fully automated system, all titration samples were serially diluted from 1:40 to 1:320, screened, and reported with intensity. After introducing the Helios system, the titration samples were screened at 1:80 dilution, and if positive, further dilution was done based on the automatically predicted endpoint titer, enabling us to reduce the number of wells used.

There were some limitations to our research. First, our study included a small number of specific patterns, such as nuclear dots and nuclear envelope patterns, limiting our ability to accurately assess the accuracy of the Helios system for these patterns. Second, we did not include the ENA or the patients’ disease status when confirming patterns by visual reading, as the goal of this study was to assess the level of concordance between automated results and human assessments under real-life working conditions. Evaluation of the Helios system in the context of these additional factors will be investigated in a future study. Finally, the possibility of inter-observer reading bias cannot be ruled out in a single-center study. This is supported by our analysis of the two expert reading results showing overall inter-observer agreements of 86.7% (*κ* = 0.69) for positive/negative discrimination and 85.4% for pattern classification. Therefore, a multicenter study will be required to overcome the readers’ subjectivity in a single-center study (9).

In conclusion, Helios, the fully automated ANA IFA analyzer showed good agreement in distinguishing between positive and negative results. However, it still has limitations in positive/negative discrimination, pattern recognition, and endpoint titer prediction, requiring additional validation of results by human observers. Helios provides significant advantages in routine laboratory ANA IFA work in terms of labor, time, and cost savings. We hope that upgrading and developing softwares with more reliable capabilities will allow automated ANA IFA analyzers to be fully integrated into the routine operations of the clinical laboratory.

## Data Availability Statement

The original contributions presented in the study are included in the article/**Supplementary Material**. Further inquiries can be directed to the corresponding author.

## Ethics Statement

The studies involving human participants were reviewed and approved by Institutional Review Board of Chonnam National University Hospital (IRB CNUH-2019-304). Written informed consent for participation was not provided by the participants’ legal guardians/next of kin because: the IRB-CNUH waived the requirement for informed consent, due to the nature of this study.

## Author Contributions

H-WC, YJK, and S-JK designed the study. H-WC and YJK generated the data. H-WC, YJK, J-HP, S-YL, SC, JHL, H-JC, M-GS, J-HS, and S-JK analyzed and discussed the data. H-WC, YJK, and S-JK ran the statistical analysis. H-WC, YJK, and S-JK wrote the manuscript. All authors contributed to the article and approved the submitted version.

## Funding

This study was supported by the National Research Foundation of Korea (2020R1C1C1007297) and the Chonnam National University Hospital Biomedical Research Institute (BCRI19024). The funding organizations played no role in the design of study, choice of enrolled patients/specimens, review and interpretation of data, preparation of manuscript, or final approval of manuscript.

## Conflict of Interest

The authors declare that the research was conducted in the absence of any commercial or financial relationships that could be construed as a potential conflict of interest.
