# Supplementary material for: Evaluation of a Fully Automated Antinuclear Antibody Indirect Immunofluorescence Assay in Routine Use
Source: Front Immunol. 2020 Dec 4;11:607541. doi: 10.3389/fimmu.2020.607541 (PMC7746920; doi:10.3389/fimmu.2020.607541)
Supplement: Supplementary file 1 [file DataSheet_1.docx]

**Supplementary TABLE 1.** Agreement between automated and classical endpoint titers for homogeneous pattern^a^.

| Classical endpoint titer | *n* |  | Automated endpoint titer, *n* | | | | | | | Agreement^b^ (%) |
| --- | --- | --- | --- | --- | --- | --- | --- | --- | --- | --- |
|  |  |  | 1:80 | 1:160 | 1:320 | 1:640 | 1:1,280 | 1:2,560 | 1:5,120 |  |
| 1:80 | 10 |  | **9**^c^ | **1** | - | - | - | - | - | 100 |
| 1:160 | 10 |  | **4** | **6** | - | - | - | - | - | 100 |
| 1:320 | 19 |  | - | **14** | **1** | **2** | 2 | - | - | 89.5 |
| 1:640 | 6 |  | - | - | - | - | **3** | 1 | 2 | 50.0 |
| 1:1,280 | 2 |  | - | - | - | - | - | - | 2 | 0 |
| 1:2,560 | 3 |  | - | - | - | - | **1** | - | **2** | 100 |
| 1:5,120 | 1 |  | - | - | - | - | - | - | **1** | 100 |
| Total | 51 |  | 13 | 21 | 1 | 2 | 6 | 1 | 7 | **86.3^d^** |

^a^Only samples with the same single pattern by both Helios and visual readings were included.

^b^Included the results within ±one titer difference which were deemed concordant.

^c^Number of the concordant results are emphasized in bold.

^d^*κ* = 0.22, reflecting only samples with the same titer between automated and classical endpoint titers.

*n*, number.

**Supplementary TABLE 2.** Agreement between automated and classical endpoint titers for speckled pattern^a^.

| Classical endpoint titer | *n* |  | Automated endpoint titer, *n* | | | | | | | Agreement^b^ (%) |
| --- | --- | --- | --- | --- | --- | --- | --- | --- | --- | --- |
|  |  |  | 1:80 | 1:160 | 1:320 | 1:640 | 1:1,280 | 1:2,560 | 1:5,120 |  |
| 1:80 | 15 |  | **15**^c^ | **-** | - | - | - | - | - | 100 |
| 1:160 | 12 |  | **7** | **5** | - | - | - | - | - | 100 |
| 1:320 | 12 |  | - | **9** | **2** | **1** | **-** | - | - | 100 |
| 1:640 | 19 |  | - | 9 | **6** | **1** | **2** | **-** | 1 | 47.4 |
| 1:1,280 | 18 |  | - | 5 | 5 | **3** | **2** | **1** | 2 | 33.3 |
| 1:2,560 | 10 |  | - | - | 1 | 2 | **3** | **-** | **4** | 70.0 |
| 1:5,120 | 20 |  | - | 1 | - | 1 | 3 | **6** | **9** | 75.0 |
| Total | 106 |  | 22 | 29 | 14 | 8 | 10 | 7 | 16 | **71.7^d^** |

^a^Only samples with the same single pattern by both Helios and visual readings were included.

^b^Included the results within ±one titer difference which were deemed concordant.

^c^Number of the concordant results are emphasized in bold.

^d^*κ* = 0.21, reflecting only samples with the same titer between automated and classical endpoint titers.

*n*, number.

**Supplementary TABLE 3.** Agreement between automated and classical endpoint titers for centromere pattern^a^.

| Classical endpoint titer | *n* |  | Automated endpoint titer, *n* | | | | | | | Agreement^b^ (%) |
| --- | --- | --- | --- | --- | --- | --- | --- | --- | --- | --- |
|  |  |  | 1:80 | 1:160 | 1:320 | 1:640 | 1:1,280 | 1:2,560 | 1:5,120 |  |
| 1:80 | - |  | **-** | **-** | - | - | - | - | - | - |
| 1:160 | 1 |  | **1**^c^ | **-** | - | - | - | - | - | 100 |
| 1:320 | 2 |  | 1 | **1** | **-** | **-** | - | - | - | 50.0 |
| 1:640 | 5 |  | - | 4 | **-** | **-** | **1** | - | - | 20.0 |
| 1:1,280 | 6 |  | - | 2 | - | **-** | **1** | **1** | 2 | 33.3 |
| 1:2,560 | 3 |  | - | - | - | - | **2** | **1** | **-** | 100 |
| 1:5,120 | - |  | - | - | - | - | - | **-** | **-** | - |
| Total | 17 |  | 2 | 7 | 0 | 0 | 4 | 2 | 2 | **47.1^d^** |

^a^Only samples with the same single pattern by both Helios and visual readings were included.

^b^Included the results within ±one titer difference which were deemed concordant.

^c^Number of the concordant results are emphasized in bold.

^d^*κ* = -0.12, reflecting only samples with the same titer between automated and classical endpoint titers.

*n*, number.

**Supplementary TABLE 4.** Agreement between automated and classical endpoint titers for nucleolar pattern^a^.

| Classical endpoint titer | *n* |  | Automated endpoint titer, *n* | | | | | | | Agreement^b^ (%) |
| --- | --- | --- | --- | --- | --- | --- | --- | --- | --- | --- |
|  |  |  | 1:80 | 1:160 | 1:320 | 1:640 | 1:1,280 | 1:2,560 | 1:5,120 |  |
| 1:80 | 3 |  | **3**^c^ | **-** | - | - | - | - | - | 100 |
| 1:160 | 1 |  | **1** | **-** | - | - | - | - | - | 100 |
| 1:320 | 6 |  | 2 | **3** | **-** | **-** | 1 | - | - | 50.0 |
| 1:640 | 1 |  | - | - | 1 | - | **-** | - | - | 100 |
| 1:1,280 | 1 |  | - | - | - | - | **1** | - | - | 100 |
| 1:2,560 | - |  | - | - | - | - | **-** | **-** | **-** | - |
| 1:5,120 | - |  | - | - | - | - | - | - | **-** | - |
| Total | 12 |  | 6 | 3 | 1 | - | 2 | - | - | **75.0^d^** |

^a^Only samples with the same single pattern by both Helios and visual readings were included.

^b^Included the results within ±one titer difference which were deemed concordant.

^c^Number of the concordant results are emphasized in bold.

^d^*κ* = 0.17, reflecting only samples with the same titer between automated and classical endpoint titers.

*n*, number.

**Supplementary TABLE 5.** Agreement between automated and classical endpoint titers for cytoplasmic pattern^a^.

| Classical endpoint titer | *n* |  | Automated endpoint titer, *n* | | | | | | | Agreement^b^ (%) |
| --- | --- | --- | --- | --- | --- | --- | --- | --- | --- | --- |
|  |  |  | 1:80 | 1:160 | 1:320 | 1:640 | 1:1,280 | 1:2,560 | 1:5,120 |  |
| 1:80 | 1 |  | **-** | **1** | - | - | - | - | - | 100 |
| 1:160 | 5 |  | **4**^c^ | **1** | - | - | - | - | - | 100 |
| 1:320 | 1 |  | - | **1** | **-** | **-** | - | - | - | 100 |
| 1:640 | - |  | - | - | **-** | **-** | **-** | - | - | - |
| 1:1,280 | 1 |  | - | - | - | **-** | **1** | **-** | - | 100 |
| 1:2,560 | 2 |  | - | - | - | - | **1** | **-** | **1** | 100 |
| 1:5,120 | 2 |  | - | - | - | 1 | - | **-** | **1** | 50.0 |
| Total | 12 |  | 4 | 3 | 0 | 1 | 2 | 0 | 2 | **91.7^d^** |

^a^Only samples with the same single pattern by both Helios and visual readings were included.

^b^Included the results within ±one titer difference which were deemed concordant.

^c^Number of the concordant results are emphasized in bold.

^d^*κ* = 0.09, reflecting only samples with the same titer between automated and classical endpoint titers.

*n*, number.

**Supplementary TABLE 6.** Characteristics of 8 SLE samples within the cohort with false negative results by Helios reading.

| No. | Sex | Age, year | Test request | Pattern | Titer | ENA^a^ | Diagnosis | Treatment |
| --- | --- | --- | --- | --- | --- | --- | --- | --- |
| 358 | F | 42 | Titration | Nuclear fine speckled (AC-4) | 1:80 | Negative | SLE with glomerular disease | observation |
| 377 | F | 53 | Titration | Punctate nuclear envelope (AC-12) | 1:160 | Negative | SLE with other organ or systemic involvement | observation |
| 449^b^ | M | 42 | Titration | Nuclear fine speckled (AC-4) | 1:80 | dsDNA | SLE with nephritis | DMARDs medication,  Hx of cyclophosphamide CTx |
| 974 | M | 44 | Titration | Nuclear coarse speckled (AC-5) /  Few nuclear dots (AC-7) | 1:160 | dsDNA | SLE, unspecified | DMARDs medication |
| 1618^b^ | M | 42 | Titration | Nuclear fine speckled (AC-4) | 1:80 | dsDNA | SLE with nephritis | DMARDs medication,  Hx of cyclophosphamide CTx |
| 1637 | F | 30 | Titration | Nuclear dense fine speckled (AC-2) | 1:160 | Negative | SLE, unspecified | observation |
| 2487 | F | 63 | Titration | Homogeneous (AC-1) | 1:80 | Negative | SLE, unspecified | observation |
| 2567 | F | 59 | Titration | Few nuclear dots (AC-7)/  Cytoplasmic reticular/AMA (AC-21) | 1:80 | U1-RNP | SLE with glomerular disease | observation |

^a^Checked for Scl-70, Jo-1, centromere, dsDNA, nucleosome, histone, ribosomal P, Sm, U1-RNP, SS-A/Ro60, SS-B/La, β2GP1, cardiolipin IgG/IgM.

^b^No.449 and No.1618 are samples from the same patient requested with an interval of one month.

β2GP1, beta-2-glycoprotein 1; CTx, chemotherapy; DMARDs, disease modifying antirheumatic drugs; dsDNA, double-stranded DNA; ENA, extractable nuclear antigen; Hx, history; and SLE, systemic lupus erythematosus.
